# CVD Prevention Through Policy: a Review of Mass Media, Food/Menu Labeling, Taxation/Subsidies, Built Environment, School Procurement, Worksite Wellness, and Marketing Standards to Improve Diet

**DOI:** 10.1007/s11886-015-0658-9

**Published:** 2015-09-14

**Authors:** Ashkan Afshin, Jose Penalvo, Liana Del Gobbo, Michael Kashaf, Renata Micha, Kurtis Morrish, Jonathan Pearson-Stuttard, Colin Rehm, Siyi Shangguan, Jessica D. Smith, Dariush Mozaffarian

**Affiliations:** Friedman School of Nutrition Science and Policy, Tufts University, 150 Harrison Ave, Boston, MA 02111 USA; Department of Public Health and Policy, University of Liverpool, Liverpool, UK; Department of Nutrition, Harvard T.H. Chan School of Public Health, Boston, MA USA

**Keywords:** Diet, Policy, Advertising, Tax, Subsidy, Schools, Regulation, Worksite, Labeling

## Abstract

Poor diet is the leading cause of cardiovascular disease in the USA and globally. Evidence-based policies are crucial to improve diet and population health. We reviewed the effectiveness for a range of policy levers to alter diet and diet-related risk factors. We identified evidence to support benefits of focused mass media campaigns (especially for fruits, vegetables, salt), food pricing strategies (both subsidies and taxation, with stronger effects at lower income levels), school procurement policies (for increasing healthful or reducing unhealthful choices), and worksite wellness programs (especially when comprehensive and multicomponent). Evidence was inconclusive for food and menu labeling (for consumer or industry behavior) and changes in local built environment (e.g., availability or accessibility of supermarkets, fast food outlets). We found little empiric evidence evaluating marketing restrictions, although broad principles and large resources spent on marketing suggest utility. Widespread implementation and evaluation of evidence-based policy strategies, with further research on other strategies with mixed/limited evidence, are essential “population medicine” to reduce health and economic burdens and inequities of diet-related illness worldwide.

## Introduction

**Fig. 1:**
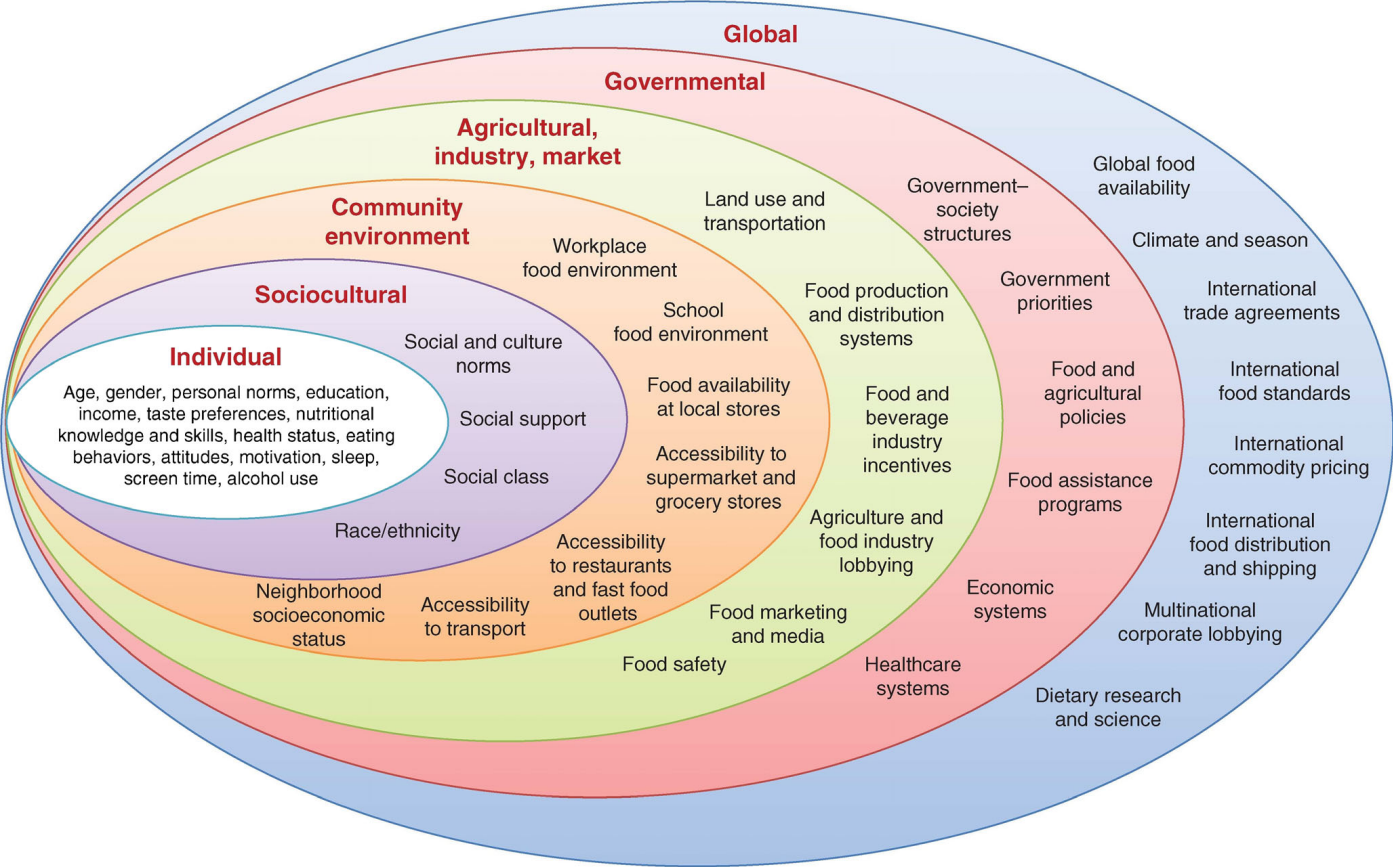
Barriers and opportunities for healthy eating. (Reproduced with permission from: Afshin A, Micha R, Khatibzadeh S, Schmidt L, Mozaffarian D. Dietary policies to reduce noncommunicable diseases. In: Brown G, Yamey G, Wamala S, editors. *The Handbook of Global Health Policy*. Wiley-Blackwell, San Francisco, 2014) [10]

Poor diet is the leading cause of cardiovascular disease (CVD), total mortality, and morbidity in the USA and globally [1, 2]. Current intakes of major dietary risk factors are suboptimal across the world [3–6]. Clearly, improving dietary habits across populations is a major clinical and policy priority of our time. While individual-level and health care system-based behavioral change efforts can be partly effective [7, 8], policy changes at organizational, community, and government levels can have broader, more equitable, and more sustainable impact [9••]. Because dietary choices are influenced by a range of determinants at individual, sociocultural, community, national, and global levels, potential policy strategies can be applied across a range of different domains (Fig. 1) [10]. To better understand the current evidence for different policy levers, we systematically reviewed the evidence for effectiveness of specific policies to improve dietary habits and reduce cardiovascular and metabolic risk factors.

## Methods

We searched multiple online databases for studies evaluating the effectiveness of policy strategies to improve diet. Specific search terms and strategies are available from the authors upon request. We focused on policies in several domains with prior evidence for effectiveness [9••], including mass media campaigns, food and menu labeling, taxation and subsidies, local built environment, school procurement policies, worksite wellness programs, and marketing standards. For certain domains (e.g., worksite wellness programs), we focused on interventional (randomized, quasi-experimental) studies; for other domains (e.g., taxation and subsidies), we evaluated both interventional and prospective cohort studies. For built environment and studies of price elasticity of demand for food and nonalcoholic beverages, we also included cross-sectional studies given the preponderance of such evidence in these domains.

Our endpoints of interest include changes in dietary habits or diet-related risk factors for cardiovascular disease, in particular adiposity, blood pressure, and blood lipid levels. To provide contemporary evidence, studies were excluded if published prior to 1980, not reporting effect sizes of the potential intervention of interest, or conducted in special populations having major underlying disease (e.g., metastatic cancer). For final inclusion decisions, all studies were reviewed and evaluated by two investigators independently. For each policy, one investigator took the lead on summarizing the evidence on effectiveness of each policy, with input and guidance from all investigators.

## Results

### Mass Media Campaigns

Mass media campaigns, either alone or as a part of a multicomponent intervention, provide a tool for the dissemination of evidence-based dietary targets. Over the past three decades, mass media campaigns have been utilized as one feature of successful multicomponent interventions targeting dietary habits at community [11–17] and national levels [18–24]. In such multifaceted interventions, the independent contribution of the mass media component is difficult to evaluate.

Several studies suggest potential effectiveness of mass media campaigns as a stand-alone intervention. These have shown temporal improvements in consumption of specific dietary factors, especially increased fruits and vegetables and (less commonly) reduced salt [25–30]. For example, after implementation of the Australian “2 Fruit ‘n’ 5 Veg Every Day” campaign (1992–1994), annual surveys found an increase in intakes of fruits (from 1.5 to 1.7 servings/day; *P* < 0.05) and vegetables (from 2.6 to 3.1 servings/day; *P* < 0.001) among adults [25]. This campaign consisted of intensive bursts of TV commercials over a 3-week period in 1992 and 1993 and a 1-week period in 1994, as well as additional print and radio ads. Based on ecologic follow-up over time, the full effect of the campaign was observed during the first year of implementation and sustained, without further increase, in ensuing years [25]. Based on nationally representative cross-sectional surveys in 1991 and 1997 following the launch of the US “5-A-Day” campaign in 1991, the proportion of US adults consuming at least 5 servings/day of fruits and vegetables significantly increased; mean national intakes also increased, although this latter finding only remained significant among nonsmokers and Hispanics after adjustment for demographic shifts [31]. Awareness of the health benefits of fruits and vegetables and knowledge of the campaign also increased during this time period [31]. Yet, although the campaign continued through 2007, a relatively low rate of awareness of this campaigns (29 %) raised concern over full coverage and penetration.

Other mass media interventions in Pakistan (1999–2004) and the USA (1995) used prominent newspaper articles and computer-based newsletters, respectively, to target fruits and vegetables [27, 28]. Of the 75 % of Pakistani readers who read the articles, 40 % reported having changed their dietary behaviors [27]; while in the USA, fruit and vegetable consumption increased (from 3.4 to 4.1 servings per day, *P* < 0.002) after 6 months of computer-based newsletters [28]. In a meta-analysis of five randomized and nonrandomized interventions from the USA, New Zealand, and Australia, including several of the studies above, implementation of mass media campaigns was associated with 0.25 serving/day increased consumption of fruits and vegetables (95 % CI = 0.15–0.35) [29].

In the UK, a national campaign targeting salt intake was associated with a reduced proportion of adults who reported adding salt at the table, from 32.5 % in 2003 to 23.2 % in 2007 [30]. This reduction was consistently observed across age, sex, and socioeconomic groups.

In sum, several studies suggest that a focused mass media campaign, targeting a single dietary factor or related dietary factors, can improve diet. The effectiveness of mass media campaigns on dietary targets beyond fruits, vegetables, or salt is not established. In addition, the quasi-experimental designs of most of these studies suggest but preclude strong conclusions about cause -and- effect of the campaigns. Further investigation is also needed on the influence of varying intensity, penetration, and duration of mass media campaigns, as well as cost-effectiveness and effects on disparities relative to other policy strategies.

### Food and Menu Labeling

Food and menu labeling approaches are widely utilized with the aim to influence consumer choice. These can take several forms including (a) nutrition panels (e.g., Nutrition Facts), (b) nutrient content claims (e.g., “low sodium,” “fat-free”), (c) health-related claims, (d) logos based on nutrition criteria (e.g., “green Keyhole” in Sweden [32], “Choices” logo in The Netherlands [33], American Heart Association “Heart-Check” [34]), (e) front-of-pack icons based on a grading system (e.g., “traffic light” icon in UK and Ireland [35, 36], “Guiding Star” program in the USA), [37] and (f) menu labeling (e.g., total calorie content) [38–40].

The effectiveness of food labeling has been evaluated in natural experiments of mandatory food labeling laws over periods from months to more than a year [41–43] and in shorter term randomized and nonrandomized interventions over a meal [44–46] or several weeks [47, 48]. Most studies were conducted in university [49, 50], worksite [47, 51, 52] or community settings [44, 46, 53] in high-income countries in North America, Europe, or Oceania, and most evaluated populations in which young and middle-aged adults, in particular females, had greater representation. Evaluation of effectiveness has been based on changes in sales [37, 46, 54], self-reported dietary intake [44, 52, 53], or diet-related risk factors (e.g., BMI) [47, 55].

Overall, these studies have not shown consistent effects on consumer behavior. One preliminary meta-analysis of interventional trials and prospective studies found no significant effect of food and menu labeling on sales or consumption, regardless of label format, dietary target (e.g., calorie content, total fat, dietary fiber), target population, food establishment setting, or mandatory vs. voluntary nature of labeling [56]. The most common dietary targets were energy intake (*n* = 23 studies), total fat (*n* = 8), and saturated fat (*n* = 4). Fewer studies have evaluated food labels and diet-related outcomes, such as trans fat concentrations in human breast milk following Canada’s trans fat labeling regulation [55] and BMI and serum triglycerides in a US worksite intervention incorporating food labeling [47].

While overall effects of food and menu labeling are not clearly identified in populations, concerns have been raised that such interventions, if effective, could potentially exacerbate disparities by having larger effects among higher socioeconomic groups compared with lower socioeconomic groups [57, 58]. Based on this concern, food labeling should be considered in combination with other intervention such mass media campaigns and education [37, 59], neighborhood environmental change [54], price incentives [52, 60, 61], and quality standards [59] to enhance effectiveness in disadvantaged populations and reduce potential widening of disparities.

Several studies have performed time-series analyses of products to assess the potential impact of food and menu labeling on industry formulations. These reports suggest that menu labeling for sodium in chain restaurants [62, 63] and front-of-package supermarket labels [64, 65] may modestly lower sodium contents [62, 64]. Further studies are needed to confirm the presence and magnitude of this effect as well as evaluate other dietary targets.

### Taxation and Subsidies

Food prices are important determinants of dietary choices [9••, 66]. Proactive strategies can include taxation to reduce intakes of unhealthful foods and subsidies to promote consumption of healthful foods [67]. Evidence on effectiveness of such pricing strategies generally derives from cross-sectional economic models estimating the price elasticity of demand for foods [68, 69•]; and a smaller number of interventional or prospective observational studies evaluating price and consumption changes over time [70].

Cross-sectional demand models demonstrate inverse relationships between price of a given food and its consumption, with magnitudes of elasticity (percent change in consumption per percent change in price) that vary depending on the food as well as a person’s country and income level [68, 69•]. For example, based on a systematic review of 160 modeling studies in the USA, price elasticities varied from −0.27 for eggs (i.e., 0.27 % lower consumption per 1 % increase in price) to −0.81 for foods consumed away from home; intermediate values were seen for fish (−0.50), vegetables (−0.58), fruits (−0.70), and soft drinks (−0.76) [68]. A systematic review and meta-analysis of 136 studies from 162 countries reported an overall price elasticity of −0.70 for all food groups combined [69•]. This varied by country income, with greater elasticity in low-income countries (−0.74) than in high-income countries (−0.56), and by household socioeconomic status within countries, with greater elasticity in low-income households (−0.91) than in high-income households (−0.77). These cross-sectional price elasticity analyses cannot distinguish between potentially varying magnitudes of effects for price increases (e.g., taxation) vs. decreases (e.g., subsides) for the same food.

Prospective observational and interventional studies confirm the effectiveness of pricing strategies to improve diet and also allow direct assessment of increased vs. decreased prices. Based on our own recent review and meta-analysis of such studies, a 10 % price reduction or subsidy increases consumption of healthful foods/beverages by 14 %, while a 10 % price increase or tax reduces consumption of unhealthful foods/beverages by 7 % decrease (unpublished). Our review also suggested that combining pricing strategies with other approaches such as altering food availability enhances the effectiveness of price changes.

Recently, Mexico reported on the changes in consumption of sugar-sweetened beverages (SSBs) 1 year following institution of a one peso per liter (approximately 10 %) national excise tax that took effect on Jan 1, 2014 (www.insp.mx/epppo/blog/3659-reduccion-consumo-bebidas.html). Based on a commercial panel of household purchasing data and adjusting for pre-existing trends in SSBs and for other macroeconomic variables, SSB purchasing was reduced by 12 % by December 2014. Effects were evident across all socioeconomic groups, with largest declines (up to 17 %) in lower socioeconomic households. At the same time, purchases of other, untaxed beverages increased by 4 %, mainly due to greater sales of bottled plain water; data on tap water intake was not collected. These quasi-experimental findings, in particular the larger effect among lower socioeconomic individuals, strongly support the efficacy of pricing measures to alter population dietary habits.

### Local Food Environment

Food choices and dietary behaviors are plausibly influenced by an individual’s local food environment, e.g., presence and accessibility of supermarkets, grocery stores, convenience stores, fast food restaurants, and full-service restaurants. Availability and accessibility of these outlets have typically been studied by evaluating density (per area or capita) or distance (e.g., to home), with usual outcomes including BMI, other obesity-related outcomes, or (less commonly) specific dietary components or dietary quality [71]. In addition, some studies have explored in-store availability of foods, walkability to outlets, distance from school or worksites rather than homes, and participation in farmers’ markets or community gardens.

Nearly all prior studies of these topics have been cross-sectional. In the present review, we identified more than >150 relevant cross-sectional studies. Most evaluated multiple neighborhood characteristics, such as outlet density measures or distance from home, in relation to one or more type of food outlet. Overall, these cross-sectional studies observed inverse associations of supermarket availability with adiposity and much more mixed associations for other types of food outlets. However, the potential for reverse causation limits strong inference: One cannot determine whether dietary choices are worse in these populations due to absence of supermarkets or whether supermarkets do not succeed in these neighborhoods due to population dietary choices. Moreover, adjustments for individual-level and other neighborhood-level characteristics in some studies were minimal, raising concern for residual confounding from other factors.

Many fewer longitudinal observational or quasi-experimental studies have investigated these questions. Most were USA studies, including in adults [71–75] and children [76–79], with variable sample sizes (*n* = 350 to 28,000 participants) and durations of follow-up (1–30 years). In these prospective studies, findings have been inconsistent, with no clear associations between availability or accessibility of supermarkets or other food outlets and measures of adiposity or diet quality.

In sum, current evidence for effects of the local built food environment on diet or diet-related risk factors remains surprisingly limited. Several challenges are event, including the cross-sectional nature of most studies, the heterogeneity in metrics and definitions used to characterize the local food environment, and the diversity of influences on diet outside the home neighborhood (e.g., other influences at schools or work). Availability of transportation could also be an important effect modifier of associations but has been infrequently evaluated. Further longitudinal investigations, examining diet and diet-related outcomes before and after changes in the local food environment, are needed to better understand how the neighborhood food environment may influence adiposity, dietary quality, and other outcomes.

### School Procurement Policies

Schools can alter dietary choices of their students through procurement policies, such as standards or guidelines for purchasing of foods. We reviewed interventional (randomized or quasi-experimental) studies of school regulation and procurement policies to evaluate their effectiveness on achieving dietary change. We included multicomponent studies if these regulations were a major component of the intervention. Relevant studies were considered across three broad categories: (1) increase in availability of healthful foods and beverages, (2) standards on availability of unhealthful foods and beverages, and (3) implementation of nutrition standards for school meals.

Thirty-one interventions assessed increased availability of healthful foods and beverages, largely in cafeterias or vending machines, with average duration of 8–10 months [80–113]. Most were small-scale, local programs; national programs were identified in Norway [83–86], the UK [95], and Canada [91, 97]. Fruits and vegetables were the most common dietary targets; other targets included low-fat snacks, milk, whole grain products, and water. Interventions that aimed to increase fruit and vegetable intake appeared effective. For example, the Norwegian School Fruit Program [85] that provided one piece of fruit or carrot per student on each school day increased fruit and vegetable school intake by 0.8 servings/day after 9 months. Similarly, a local school program that distributed free fruits and vegetables for 8 months increased total fruit intake by 0.55 servings/day [90].

Twenty-six interventions evaluated the effect of restricting unhealthful foods and beverages in schools [92, 99, 101, 114–131], with average duration of follow-up of 2 years. School settings include cafeterias, vending machines, and other competitive foods such as at school stores, snack bars, or snack trucks. Types of policies included restrictions or bans, nutrient standards for competitive foods, and combinations of these approaches. About half were local programs; others were based on city, state, or national policies. Overall, the interventions appeared generally effective in reducing intake of unhealthful competitive foods and beverages and decreasing incidence and prevalence of overweight/obesity. Governmental and school policies appeared more effective compared to voluntary programs. For example, the Boston Public Schools Snack and Beverage Policy that restricted SSBs at vending and a la carte settings found a 0.3 servings/day reduction in total daily SSB intake by students at 18 months, compared to no change in national trends during this same time period [122]. In the HEALTH study, a randomized controlled trial conducted in 42 schools across five US states, a SSB ban, and limits on milk fat content and energy content of food served throughout the school environment decreased obesity prevalence by 19 % at 3 years [132]. In the randomized controlled School Nutrition Policy Initiative in 10 US schools, an intervention that banned SSBs and restricted milk fat content and sugar and total/saturated fat content of snacks sold throughout the school environment reduced overweight incidence by 50 % and overweight prevalence by 35 % after 2 years [116].

Twenty-two interventions assessed standards for school meals for lunch and/or breakfast [97, 125, 129, 132–155], with average follow-up of 18–24 months. Standards were typically based on both types of foods (e.g., fried potatoes) and nutrient content (e.g., total fat, saturated fat, sodium) or portion size (e.g., portion of milk). Nine of these studies were randomized trials at the local level [129, 132, 134, 136, 139, 141–143, 145–148, 153, 155], while 13 were quasi-experimental studies of governmental policies (e.g., national food-based standards for school lunches in the UK and Texas [97, 125, 140, 149–151], the US Child Nutrition and WIC Reauthorization Act [133, 154], and other programs [135, 137, 138, 144, 152]). About one third of studies combined school meal standards with food and nutrient standards for competitive foods and beverages. About half also had nondietary targets, most commonly education or strategies to increase physical activity.

Dietary targets varied appreciably; most common were total fat, saturated fat, sodium, fruits, vegetables, whole grains, SSBs, milk, and sweets. For example, the HEALTHY study aimed to lower the fat content of foods served; provide 1 serving of fruits and/or vegetables at breakfast and 2+ at lunch; limit calories of dessert and snack foods to up to 200 kcal per item; limit beverages to water, 1 % or skim milk, and 100 % fruit juice (6 oz limit, only at breakfast or as an after-school snack); and provide 1 serving of fiber-rich grain-based food at breakfast and 2+ at lunch [132, 139, 148].

Overall, results were inconsistent across studies of school meal standards, with some but not others demonstrating intended changes in diet or diet-related risk factors (e.g., BMI). For example, one national regulation with food and nutrient/portion size standards found a decrease in student BMI [133], while other similar programs did not [97, 132, 134, 138, 153]. The relevance of the primary dietary targets in these analyses may influence efficacy; for example, focusing on total fat or other single nutrient targets may have little impact. No consistent patterns were identified to explain the heterogeneous findings, e.g., according to type of policy, dietary target, setting, etc. In sum, changes in school procurement policies appear effective for either increasing healthful or reducing unhealthful choices, while setting of nutrition standards have less consistent benefits.

### Worksite Wellness Programs

Most adults spend much of their weekday at work, making it a natural setting for health promotion strategies. Worksite wellness interventions typically focus on improving health, reducing insurance costs, and increasing productivity. Programs have used a variety of mechanisms to promote health and can be directed toward the entire workforce or high-risk individuals.

Prior reviews have concluded that multicomponent worksite wellness programs targeting diet, physical activity, and tobacco appear effective for improving employee health [9••]. We reviewed these prior reports as well as more recent interventional studies targeted toward the general employee population that included an external control group and evaluated changes in diet, adiposity, or CVD risk factors. Durations of interventions were highly variable, ranging from weeks to several years. Typical components included employee steering committees; group education classes; promotional and educational materials such as newsletters, signs, and brochures; health risk assessments; weight loss competitions; group exercise classes; signs to promote stair use; and cafeteria changes such as increased availability of healthy foods and nutrition labeling.

We found that several, although not all [156–164], worksite programs improved employees’ diets, especially fruit and vegetable intake [165–168], and reduced adiposity [169–172]. For example, a 2-year multicomponent worksite intervention increased fruit and vegetable consumption by 0.3 servings/day, which was maintained for 2 years post-intervention [166]. A 2-year worksite intervention among teachers resulted in 3 lb weight loss [169]. Other recent systematic reviews found evidence that worksite wellness interventions reduce body weight [173] and increase fruit and vegetable intake [174].

In sum, comprehensive worksite wellness interventions appear effective at improving diet and diet-related risk factors. Yet, while worksite wellness programs have been tested with controlled interventions, certain methodologic challenges limit strong inference from some of these studies. These include heterogeneous intervention strategies, small sample sizes, short durations of follow-up, incomplete quality of reporting, moderate loss to follow-up, and low participation rates. Future research should be directed toward optimizing targets of interventions (e.g., food-based healthful diet patterns [175]), understanding optimal intervention components and their interactions (e.g., peer support, competitions, environmental change, mobile device feedback), increasing employer and employee participation and retention, increasing duration of interventions and follow-up, and evaluating programs in low- and middle-income nations.

### Marketing Restrictions and Quality Standards

Based on the recognized power of marketing and billions of dollars spent by industry, implementing quality standards or restrictions on advertising of foods to youth is widely considered an effective strategy to improve diet [9••, 176, 177]. US children aged 2–5 and 6–11 years are estimated to view 10.9 and 12.7 food-related television advertisements each day, respectively, most often for foods and beverages of limited nutritional value [178]. Yet, despite compelling evidence that marketing of less healthful foods/beverages to children is common and associated with preferences and purchase requests, there is little data quantifying the impact of marketing standards or restrictions on long-term dietary intakes or health endpoints. In a longitudinal study of US children, based on indirect assessment of exposure to local fast food advertising, a theoretical complete ban was estimated to reduce the prevalence of childhood overweight by 18 and 14 % among 3–11 and 12–18-year-olds, respectively [179]. These single estimates, without reported measures of precision, led to suggestions that food advertising bans are a highly cost-effective approach to reducing global chronic disease [180]. While there is little doubt that marketing impacts dietary preferences and purchase requests and while other experiences (e.g., tobacco control) strongly support benefits of marketing standards or restrictions, evidence to determine potential magnitudes of benefit for diet and adiposity remain elusive.

From legal and constitutional perspectives, placement of quality standards or restrictions on food marketing to children is supported by evidence that such advertising is deceptive, as children are unable to differentiate marketing/advertisements from content programming [177], although this theory has not been tested in US court [181]. Several countries, including Greece, Sweden, Belgium, UK, Ireland, and Quebec, have either restricted or set quality standards on how foods are marketed to children [182]. Evidence on the impact of such policies remains sparse. In the UK, statutory scheduling restrictions on the times of marketing to children of foods high in fat, sugar, or salt were more than offset by increased advertising of these products in other hours, so that overall exposure to such marketing did not change in children and substantially increased in adults [183].

In efforts to limit external regulation, industry consortia such as the Children’s Food and Beverage Advertising Initiative, spearheaded by the US Council of Better Business Bureaus and many food manufacturers/restaurants, have voluntarily committed to not advertise or only advertise approved foods to children younger than 12 years [182, 184, 185]. However, the nutrition standards of this industry-supported program have been critiqued as being lax [186]. In addition, many major food companies are not participating, and children still have relatively high levels of advertising exposure on programs aimed at adolescents and adults [183, 187].

Based on broad observations, it is evident that marketing influences food choices in both children and adults. Quality standards or restrictions on such marketing present a promising strategy for improving population choices; more studies are needed on outcomes in countries implementing such approaches. Also, as the nature of advertising has shifted from television and other traditional media toward mobile, internet, program placement, and game-based marketing, additional research is needed to evaluate the impact of quality standards on these approaches. In addition, there is little data on exposure to marketing and policy standards in low- and middle-income countries, a crucial area for future research.

## Conclusions

Our review supports the effectiveness of specific policy strategies to improve diet. These include focused mass media campaigns (especially to increase fruits and vegetables and to reduce salt), food pricing strategies (including both subsidies and taxation, with stronger effects at lower income levels), school procurement policies (to either increase healthful or reduce unhealthful choices), and worksite wellness programs (especially when such programs are comprehensive and multicomponent).

Evidence was mixed and inconclusive for efficacy of food and menu labeling (on either consumer or industry behavior), changes in the local built environment (e.g., availability or accessibility of supermarkets, fast food outlets, etc.), and implementation of nutrition standards for school meals (e.g., for school lunch or breakfast). We found little empiric evidence to evaluate effects of marketing restrictions, although broad principles and large resources spent on marketing suggest that this is a promising area for application and assessment.

Suboptimal dietary habits are now the leading cause of poor health in the USA and globally [1, 2].

Widespread implementation and evaluation of evidence-based policy strategies, as well as further research on other strategies with mixed or limited evidence, are essential approaches to “population medicine” to reduce the health and economic burdens of diet-related illness worldwide.
